# Exploring narratives of resilience among seven males living with spinal cord injury: a qualitative study

**DOI:** 10.1186/s40359-017-0211-2

**Published:** 2018-01-04

**Authors:** Anne Geard, Marit Kirkevold, Marianne Løvstad, Anne-Kristine Schanke

**Affiliations:** 1 Sunnaas, Rehabilitation Hospital, Bjørnemyrveien 11, 1450 Nesoddtangen, Nesodden, Norway; 2 Department of Nursing Science, University of Oslo, Postboks 1130 Blindern, 0318 Oslo, Norway

**Keywords:** Spinal cord injury, Thematic analyses, Adjustment processes, Long- term perspective, Resilience

## Abstract

**Background:**

It is a challenge for both individuals and families when an illness or traumatic injury results in a severe spinal cord injury. The on-going physical impairments experienced by persons with spinal cord injury play themselves out over time. Few qualitative studies have explored how health, resilience and wellbeing interplay across time among persons living with the consequences of severe physical injuries. Thus, the aim of this study was to obtain a deeper understanding of how individuals with spinal cord injury reflect upon the efforts, strategies and agency they perform to sustain long term resilience and wellbeing.

**Methods:**

In this exploratory qualitative study, we conducted a thematic analysis of in-depth interviews with seven men who had lived with spinal cord injury for 2–32 years and who previously had undergone medical rehabilitation.

**Results:**

The efforts revealed by the participants in normalising life with a spinal cord injury required continued flexibility, persistency and solution-focused adjustment, interpreted as processes documenting resilience. The participants were marshalling personal resources to handle challenges over time. They explained that they succeeded in maintaining health and wellbeing by manoeuvring between different strategies such as being self-protective and flexible as well as staying active and maintaining a positive attitude. Further, support from relational resources were of utmost importance emotionally, socially and when in need of practical assistance. When harnessing relational resources when needed, the participants underlined that balancing dependence and autonomy to remain a part of ordinary life was essential in staying emotionally stable.

**Conclusions:**

The findings of the present study show similarities to those of previous studies with regard to the participants’ attribution of their resilience and wellbeing to their innate personal abilities and strong connection to their family and friends. In addition, the current participants provide enlightening nuances and depth that expand our understanding of the construct of resilience by highlighting the importance of continuously exerting agency, willpower and strength through rational cognitive strategies to adjust and adapt to chronic and new challenges.

**Electronic supplementary material:**

The online version of this article (10.1186/s40359-017-0211-2) contains supplementary material, which is available to authorized users.

## Background

Living with the long-term consequences of severe and disabling injuries represents a significant challenge with regard to health and wellbeing for both the injured persons and their families [1–6]. One patient group that typically lives with life-long, significant and complex functional impairments comprises those who have sustained a spinal cord injury (SCI). Injuries to the spinal cord can be caused by traumatic transport accidents, falls, recreation and sports activities, or they may be non-traumatic, caused by various medical disease processes, such as infections or tumours [7]. A wide range of physical health problems can result from the sensory, motor and neurological impacts of the injury, and whether the injury is complete or incomplete affects the functional outcome [7]. The loss of mobility and sensation also results in challenges such as bladder and bowel problems, bedsores, spasticity and pain [8]. It has also been documented that physical disabilities also influence wellbeing, leading to a long-term decreased health-related quality of life and life satisfaction as well as elevated levels of post-traumatic stress symptoms and depression [5, 9–11]. Quantitative studies have additionally shown that psychosocial and occupational functioning can be greatly affected by SCI due to loss of work opportunities; changes in income, dwelling, and family life; and altered opportunities for socialising [8, 12]. Thus, there is considerable knowledge regarding the potential negative consequences of living with SCI.

During the last decades, as illustrated above, health research has primarily focused on documenting symptoms and risk factors for adverse outcomes after SCI. However, the scientific community has shifted away from establishing risk factors and negative consequences to exploring variables that enhance and promote positive processes and outcomes [13]. The field of resilience research is an example of this trend, although no overreaching theoretical frameworks of resilience exist. However, resilience refers to trajectories of positive adaptation after difficult periods. Additionally, resilience is described as a dynamic process where the effort is put into exploring a person’s capacity to “bounce back” in the face of adversity, meaning that relatively stable and healthy levels of psychosocial functioning are maintained despite traumatic life events [14–16].

Despite different research traditions similarities such as personal attributes, family support and external support systems are included qualities used to characterize resilience [17–19].

Some adult studies have demonstrated that resilient trajectories following adversity are common [14, 15, 20]. The field of resilience studies, however, has a tradition of exploring resilience as distinct outcomes following psychological trauma such as bereavement and isolated incidents. The consequences of physical injuries such as SCI, on the other hand, play themselves out over decades and interact with the on-going physical impairments experienced by the patients. As a result, this type of resilience is less studied.

Studies have highlighted that how the person copes and adapts his or her lifestyle to living with the consequences of physical injury is of greater importance for maintaining psychosocial wellbeing than the severity of the injury itself [21] and that the capacity for resilience is closely related to psychosocial wellbeing [12]. Still, few studies have explored health, resilience and wellbeing in persons with long-term physical limitations, such as SCI. White et al. [22] stated that, due to a lack of research related to patient groups for whom the traumatic event is coupled with the permanent loss of physical functioning, it is unclear whether they have the same capacity to maintain psychological wellbeing as persons who have experienced psychological trauma without physical injury. Since then, several quantitative studies have indicated that resilient trajectories are also common following physical injury such as multi-trauma and SCI [23, 24]. Furthermore, life satisfaction, low psychological distress, internal locus of control and high levels of self-efficacy correlate positively with resilience, while depression is inversely correlated to resilience [22, 25, 26]. There are few qualitative studies exploring the concept of resilience among persons with SCI. A study of twelve male rugby athletes (age 21 to 41 years) with SCI focused on the resilience process and the importance of participation in sport activities [27]. Focus group interviews with twenty-eight persons with SCI revealed that the participants attributed resilience to personal abilities, social support and the importance of being fellowmen to others with SCI [28]. A study investigated applied strategies used by persons with SCI to function autonomously, such as planning and organizing, being assertive and asking for help and learning from peers with SCI [29]. Four studies explored how persons with disabilities in general [30, 31] and SCI in particularly [32, 33] conceptualized participation and integration, and found that key elements were accessibility, social support and social connection, experiencing personal growth and feeling validated. But a major challenge was barriers in society.

In summary, recent studies have indicated that positive psychosocial wellbeing is not uncommon following SCI. Still, ambiguities exist in identifying and understanding the protective factors that enhance and promote resilient processes and positive outcomes. The present study explores what persons who have lived with SCI over time perceive as contributing positively to their long-term post-injury adjustment. Thus, the main aim of this study was to obtain a deeper understanding of how individuals with SCI reflect upon the efforts, strategies and agency they perform to sustain resilience and wellbeing from a long-term perspective. A better understanding of what persons with SCI experience as important in maintaining and fostering psychosocial wellbeing over time is pivotal knowledge for health professionals who meet the patients during acute and post-acute phase rehabilitation. Particular focus was given to what type of support the participants in hindsight regarded as important.

## Methods

The study used an exploratory qualitative research design. It was inspired by narrative theory, which highlights that open qualitative descriptions (narratives) reflect a person’s self-understanding and explain how they make their lives comprehensible and try to live up to moral demands. The narrative explanations are described as a cognitive process that gives meaning to experiences of personal action and temporality in a retrospective perspective. Further, narratives are interpreted within present knowledge and current cultural framework [34]. Additionally, the current study was inspired by Riessman [35], who emphasises that how people choose to present themselves through narratives is both a means of constructing identity and a means of coping in difficult situations across the lifespan.

### Ethics

The study was conducted according to the World Medical Association Declaration of Helsinki [36] and was approved by the Regional Committee of Medical and Health Research Ethics of Southeast Norway, (REK number 2012/1430). Participation was voluntary; the participants received written information, and they provided signed informed consent.

### Recruitment procedures and participants

When the participant is presented in the results, fictive names have been used to protect the participants anonymity.The participants were former SCI patients at a large rehabilitation hospital in Southeast Norway. This study forms part of a large qualitative study of resilience in families after severe injuries, including persons with SCI and acquired brain injuries and their close relatives. The participants were invited to participate through mailed written information and informed about the interviewers’ professional background, and work place within the hospital, as well as roles in the study.

Participants over 18 years of age who fulfilled the inclusion criteria and who had been injured for a minimum of 1.5 years were included. Participants were excluded if they were medically unstable, had major psychiatric disorders or had extensive on-going substance abuse. An invitation to participate was sent to 59 former patients, where 13 persons with SCI responded positively. Four of the participants preferred to participate in a focus group interview, and two withdrew, providing seven individual interviews, with all participants being male. The age range was 35–75 years and 2–32 years since injury; six were married or cohabitants, and one was divorced. Four of the participants had adult children and three had grandchildren. The remaining three had younger children. Three persons were permanent wheelchair users, and one was a partial wheelchair user, being able to walk for short distances. The other three participants were able to walk with some difficulty, but did not use any walking aids.

### The interviews

The interviews were obtained using an open-ended, flexible interview guide [37] (Additional file 1). The female interviewers encouraged the participants to start by telling about their pre-injury family life and then about the time of injury and hospitalisation. Next, they were invited to describe what had contributed positively during the adjustment processes post-injury. The participants were asked what influenced how they faced challenges and what was helpful when adjusting to an altered life, both during the time of injury and in the post-injury period. Finally, we asked about their thoughts regarding the future. If necessary, we probed to elicit more substantial descriptions [38]. Four individual interviews were conducted at the rehabilitation hospital and three in the participants’ homes. The interviews lasted between 45 and 88 min and were recorded on an iPod. The participants gave long content-rich interviews that covered central aspects of living with SCI, which we believe compensate for the restricted sample size of the study.

### Data analysis

Inductive thematic analysis was used to identify, analyse and describe essential themes in the participants’ narratives [39]. The analysis began by listening to the audiotapes, and then detailed verbatim transcripts were produced followed by the removal of unnecessary words, fillers and repetitions. In the second step, initial coding took place to identify meaningful units in the text of each transcript until no new information was obtained. Further the units were organized into conceptual themes that captured the meaning in each individual’s narratives. In the third step the individual themes were compared and contrasted to identify similarities and differences across the narratives. Then the themes were grouped into common themes across the interviews until further grouping of themes was no longer feasible. The thematic analysis revealed two main themes and six sub-themes further described in the result section.

## Results

Despite differences in age, life situations, severity of and time since injury, two overarching themes and six subthemes were identified in the thematic analysis. Although the themes and sub-themes are structured and described separately, they should be interpreted as on-going, mutually interactional processes.

### Marshalling personal resources to handle challenges over time

The participants’ narratives revealed important aspects of their self-understanding, their own mentality and special characteristics. Positive thinking, such as being determined to get better, taking responsibility for one’s own progress, pursuing realistic goals, and positive self-appraisals, was emphasised as important for ensuring emotional stability.

#### Maintaining a positive attitude

All participants acknowledged experiencing emotional challenges and expressed that there had been ups and downs during the time of injury and after living with a SCI. They emphasised the use of personal resources in handling these challenges, and some described an evolving capacity to deal with the situation by being stubborn or by using other strategies, such as inner dialogues and constructive self-talk, to maintain psychological wellbeing. Dean is a good example in this respect. He was paralysed within four days from the neck down due to a spinal tumour, but he regained some function in his arms and legs after several months. He believed that his stubbornness, in a positive sense, was a helpful attribute in staying focused and in maintaining his fighting spirit. Despite the vicissitudes, he told himself, “I must be able to manage this, and I will achieve my goals”. Moreover, he continued as follows:

I would not wish for my worst enemy to experience what I have experienced. I understand very well those who give up; certainly, many do. I almost gave up, too, but I said to myself, “You just have to get those thoughts out of your mind!” If you go down that road, then there will only be negativity. I told myself that I could just sit down and do nothing right away, but no, that’s not me. So, there have been many of those inner conversations.

The strategy of being cheered on by inner dialogues to maintain psychological wellbeing in the face of stressful experiences was described by several of the participants. Earl recounted how he told himself that he had actually broken his back and was paralysed from the waist down; but then, he changed his perspective: “I told myself not to focus on these negative thoughts”. In a similar manner, when Georg had a bad day, he said to himself, “I have no use for this… and I find something positive to think of”.

The participants believed that having a positive outlook on life was an innate ability or an inner strength that helped maintain psychological wellbeing. Several participants used terms such as “being an optimist” and “having a strong psyche” to describe what they considered to be basic elements that had helped them maintain psychological wellbeing. Earl explained his strong psyche in terms of rarely being emotional or depressive but, instead, dealing with his challenges and being “determined to get back on my own two feet”. The participants described “staying strong” by positive thinking, which enabled them to turn hardship into something positive. Frank, injured nearly 20 years ago, highlighted that being an optimist was the most important attribute he had. Regardless of how steep the downhill had been, he had “managed to think positively”. Several of the participants stated that having a strong psyche fostered optimism and confidence about the future. Georg considered a strong psyche as key to staying optimistic in the face of serious situations:

I have a pretty strong psyche. I’m very happy because otherwise I would not be where I am today. … There are always a few little things that make things positive. I think that if you’re able to be an optimist, you will manage to keep up with a lot of exercise and maintain a good spirit.

Georg explained metaphorically how he used his time and energy—and his strong psyche—in working through problems, even if things looked completely hopeless. He told of sometimes being “down at the basement staircase, but never further than the third step; then I turned and went back up”. These statements from the participants shed light on a common phenomenon, namely a mental capacity to defocus problems and stay focused on the positive.

To see themselves as being lucky was noted by several as a strategy that helped focusing on positive aspects of their situation. Comparisons were made with the “worse cases”, and their own limitations were downplayed. Brian felt lucky compared to those with injuries who had practical professions, due to his higher level of education and a job that was manageable despite his severe injury: “I was lucky having the background that I have when I injured myself. At least I had a job I could carry on with”. Earl also believed he had good fortune when seeing others with disabilities “who have not come close to what I have achieved”. Georg regarded himself as being lucky due to his “good genes”:

I am terribly lucky because I have some genes that are good to have when something happens. I do not take sorrows in advance, and the most important thing for a person like me who is sitting in a wheelchair is to accept that that’s the situation.

It appears that feeling lucky kept despair at a distance and supported psychological wellbeing.

#### Allowing oneself to be self-protective

The participants’ stories also contained descriptions of various self-protective strategies. To avoid exhaustion, they used coping strategies such as to withdraw from the daily demands imposed upon them due to limited capacity. They spoke of “selfishness” (i.e., focusing mainly on themselves) as a way of exerting self-protectiveness to obtain rest and having the time and energy to endure and cope during difficult times, such as periods involving hospitalisation or longstanding pain. Frank said that he was very self-protective and often thought of himself first:

I’m passionate about having to take responsibility for myself; you cannot expect anyone else to do so. I’m a selfish person when I have back pain … I disconnect and tell my wife that I am taking the car out for a spin, or shut myself in a room playing games on the computer.

#### Staying active and flexible to maintain health and wellbeing

Some explained that their success in enduring difficulties over time and maintaining psychological wellbeing without giving up was accomplished by trying to manoeuvre between different strategies. Keeping up their own interests and activities despite physical restrictions was a commonly used strategy. Albert, who experienced long periods with medical complications and mood swings that kept him immobile, said that “sometimes you have to try to make the best of the situation”. Consequently, he spent hours reading newspapers and using the Internet. When in better shape, he was a more active person driving his car, traveling abroad, and spending time with friends and family. Life was a struggle sometimes, and Dean chose to stay active by “expending a considerable amount of energy on physical training”.

The ability to be flexible and change behaviours to maintain psychosocial wellbeing despite the challenges the participants faced was evident in several interviews. Both Brian and Charles spoke of making judgments in life that involved cost-benefit trade-offs to a greater extent than non-injured people. Brian being paralysed from his neck down, described how exhausting it was to live with a disability for many years and that he had made some difficult choices to maintain his health and wellbeing:

I decided to quit my job; I was tired and had worked enough in a wheelchair, so I thought enough was enough and decided that I’m leaving. I didn’t “hit the wall”, but it was all the physical and practical challenges. … I was tired of the home care nurses arriving too late in the morning; I was tired of waiting for the car that was going to drive me to work and of looking for handicap toilets when attending work meetings, so I decided to retire.

Charles, despite his arms and legs worsening with age due to the sequelae of his spinal tumour, was still walking and clarified that “I’m pretty good at aligning myself to the situation and finding solutions that make life easier”. Despite feeling more tired after 30 years with increasingly reduced function, he emphasised that staying active was important for his wellbeing. “If manageable, I want to work for four more years, until retiring age”. Not knowing what tomorrow would bring, both Earl and Brian emphasised staying active. Earl said that, despite the vicissitudes, “I am content with life”, and that he and his cohabitant wanted to live out their dreams straight away and to “experience what’s possible to experience so we can look back on a rich life”. Brian envisioned investing within the next ten years because he felt that age took its toll with his severe injury and that he had to act when he still had the health to do so:

I’m in a wheelchair and will not become any better. There is nothing to get excited about that we are getting older, so if we are to have a nice time, it must be over the next 10 years, where she and I can have a good time and do things together.

### Staying connected and accepting help when needed

In the process of adjusting to life with SCI and maintaining psychosocial wellbeing, important others, such as rehabilitation staff, peers, family and friends, were highlighted as important resources. They were spoken of as “mentors”, “stabilisers”, “supporters”, “role models” and as an integral part of adjusting to changes in life. The fact that other people were engaged in the participants’ emotional wellbeing and physical health by encouraging progress and providing sustained support was described to be of great significance.

#### Harnessing relational resources when needed

Family members were highlighted as having an essential position in the participants’ lives, providing emotional support and practical assistance during the hospitalisation and thereafter. Earl emphasised the significance of his cohabitant staying at the acute and rehabilitation hospital as “absolutely fantastic” and that it had bearing on his “willingness and motivation to fight”. Family unity was illustrated by describing family as a team supporting each other and sharing a perception of togetherness. Albert said that “my wife and I have handled it well together” and Georg felt that “things fall into place” when he and his wife use humour consciously as a shared psychosocial stabilising strategy. Children, stepchildren and grandchildren were also highlighted as an important impetus to the participants’ wellbeing. Dean expressed that what supported his progress was the thought of his family. “It’s my two children who drive me forward”.

In addition to family, support and encouragement from the rehabilitation team was described as important. Georg’s primary nurse was a tough mentor who challenged his limits: “She could sit and watch that I almost fell off my chair when trying to get somewhere”. Frank also reported the importance of being supported by the rehabilitation staff and of being in a safe place during rehabilitation. The support promoted his self-confidence and psychological wellbeing when initially experiencing a time of uncertainty.

After an injury, you do not know where you end up in life… you feel that you are treading in a huge swamp. … Then it is important that someone helps you out of the swamp. …Learning to do everything on my own when living in an apartment inside the hospital built up my confidence, so I dared to go home.

Some of the participants found it rewarding to socialise with peers at the spinal cord unit, sharing knowledge and the mutual experience of being injured. For some, this also led to new friendships. Events organised by the Spinal Cord Injury Association were seen by some as an important meeting place that contributed to improved physical functioning and psychosocial wellbeing. Albert stated that “you get many tips on wheelchairs and traveling destinations that are accessible, and you meet a lot of very positive people, and that means a lot”. Georg had experienced that the community with peers was one way of dealing with the psychosocial stressors he encountered, where humour and laughter kept his spirits up and boosted his motivation:

The patients whom I met in rehabilitation were not in their right minds; they had a “sick” sense of humour—a kind of black humour. My, what fun we had, and it strengthened all of us to keep on fighting.

Another stabilising element during the time of injury, and in everyday life post-discharge, was emotional and practical support from friends. Brian had known many of his friends for years and told of many activities they did together both before and after his injury. He had always been a social person with many friends, and to maintain “my friendships, I prioritised spending a lot of time keeping in touch”.

#### Balancing dependence and autonomy to stay part of ordinary life

Activities the participants regarded as part of a normal life with family and friends were highlighted as important and contributing to psychosocial wellbeing. When being praised by people because he had worked and managed himself well, Brian attributed this to the fact that he was living up to the mainstream values of normalcy. “I’ve had girlfriends, cars, and a house and managed like everyone else”. A shared notion expressed by the participants was the importance of being as self-driven as possible to avoid being perceived to be a burden. Georg was “looking for practical solutions that would enable me to manage on my own as much as possible”. When his friends offered help, “I tell them thank you, but I’ll have to see. I have to try managing on my own”. He was ambivalent towards receiving help when actually being in need of it. “They carry me up the stairs. I hate it, but at the same time, if I want to participate, I have to”. This duality can be seen as a conflict between the wish to be independent and being part of social life, which sometimes implied dependence on help. Another dilemma was highlighted by Charles, who stressed that it is important not to be too much of a burden by putting too much responsibility for their own wellbeing on others:

One has to be aware of not making life a living hell for those around you; when you are in such a situation, you suddenly find yourself with a limited network.

Brian consciously urged family members to pursue their own interests or attend social events without him. He did not want his cohabitant to become bored with him, so “I challenge her to do things that I cannot attend with friends and family”. He had a strong desire to be independent, and the things he could do on his own gave life meaning, such as “meeting friends, surprising people, and planning events for family and friends”. He also travelled alone with his drive aggregate connected to his wheelchair:

I can drive where I want to with my scooter; I go to the cinema, theatre and concerts. I run across the city and meet friends, I’m an extreme outdoors person and can go far with my scooter, and then I’m totally free in a way.

#### Handling the lack of accessibility in society

Restricted accessibility in society for people with movement limitations or in wheelchairs was highlighted as frustrating, as well as more challenging with increasing age. Reduced walking ability interfered with everyday life. Albert was “frustrated due to the lack of accessibility in many of the places I want to travel”. Georg told how being physically restrained had psychosocial consequences when “some things are a bit unmanageable, I say no thanks to social events, and that is not a good feeling”. Brian was tired of being carried, and sidewalks and stairs annoyed him more than before:

In my 27 years in a wheelchair, the facilitation by society for wheelchair users haven’t improved, and it makes me disappointed and more annoyed than before. I do not know if it’s because I’ve grown older, or if it has to do with the disability, but I think it has been harder to be a wheelchair user for the last 10 years.

## Discussion

The participants in the present study illustrate important aspects of how persons with serious injury from a SCI try to exert agency by employing rational cognitive strategies to make the best of a severely altered life situation. This is an original finding of interest that has not previously been explored in detail in qualitative studies among persons with SCIs. The participants described moments and periods of being overwhelmed and distressed when faced with the long-term consequences of SCI, like other persons faced with severe injuries. However (and not previously studied in detail), the participants described the great effort willpower and strength required to counteract negative feelings and negative attributions to “bounce back” and regain emotional control. The participants described how they dealt with challenges by having inner dialogues with positive self-talk to preserve a positive outlook and maintain emotional stability. In addition, the participants highlighted their engagement in various activities such as training, visiting friends, travelling and sports activities, to maintain an ordinary life. These findings illustrate the effort taken in exerting personal strength and competence to promote psychosocial wellbeing. In addition, the participants underlined the role played by important others [5, 26], which is consistent with definitions of resilience as a multifaceted construct, reflecting personal, psychosocial and contextual processes [14, 17]. However, in addition, the current study underlines the importance of cultivating the capacity for resilience, rather than regarding it as inborn ability or stable personality trait. Furthermore, the participants spoke of making decisions successively by analysing the cost–benefits and identifying priorities to a greater extent than before. This is a core theme among persons with disabilities due to their physical limitations [40].

Several themes identified imply that the participants perceived that they exerted agency and made practical, emotional and cognitive use of the personal abilities and relational resources available to them, such as staff members during rehabilitation, as well as family, friends and peers. The process of employing both personal abilities and external support was described as intertwined and reciprocally influencing each other. This finding is in line with previous literature, underlining that personal, social and structural dimensions, such as accessibility and health services, contribute to adjustment processes and resilience [18, 27, 28].

Unlike studies that have mainly focused on resilience as a distinct outcome [15, 24], the personal experiences in the present study illuminate adjustment processes across time. The findings of the present study have traits in common with the current literature on adjusting to SCI, emphasising that positive thinking, being optimistic, believing in oneself and maintaining a positive attitude can strengthen adjustment processes and emotional stability [25–28]. In addition, the participants emphasised that their ability to face challenges evolved and changed over time. On the other hand, they demonstrated that they alternated between strategies when needed, confirming the findings of deRoon-Cassini et al. [21] and studies exploring situational coping [41, 42]. Additionally, they described the importance of exerting flexibility. This finding is in line with Bonanno et al. [43], who highlighted mental flexibility as an important part of the resilience concept.

Some studies have focused on the strain an injury imposes on family life after SCI [1, 2, 6]. In the present study, the participants conveyed a general impression of togetherness within the family and described how their social relations had different roles such as mentors, stabilisers, supporters, practical helpers and role models. It was evident that peers played a significant role in providing support, which is supported by the findings of Ljungberg et al. [44]. Being a role model to others was likewise important, as highlighted by Monden et al. [28]. Friends were described as stabilisers and important supporters to maintain psychosocial wellbeing. Strong wishes to be independent, findings which are in line with several studies [29–33], and contribute within the family, socially and vocationally and at the same time not being a burden to family members or friends was expressed.

The participants in this study have given examples that could be informative for the rehabilitation setting. Family members were highlighted as having an essential position in the participants’ lives, both with regard to emotional support and practical assistance throughout the adaptation process. This finding strongly illustrates the importance of involving family members in the rehabilitation by the interdisciplinary team. For example, Earl emphasised how important it had been to his treatment motivation that his cohabitant could stay at the hospital. Children, stepchildren and grandchildren were also highlighted as having significance. In addition to family, support from the rehabilitation team itself was described as important, both through emotional support and in providing functional challenges in safe surroundings. Some of the participants found it rewarding to socialise with peers at the spinal cord unit, as they all shared the existential experience of having an injury and were also able to trade practical advice. Events organised by the Spinal Cord Injury Association played the same role. Another stabilising element was emotional and practical support from friends and the possible role of friends in the rehabilitation process was shown.

### Limitations and strengths

A limited sample of those invited to participate responded. Thus, the current participants are likely not representative of persons living with SCI in general. We were primarily interested in how the participants described their experiences with living with SCI over time, seen from their current position in the course of the injury. Howevere, we acknowledge that their narratives will be colored by their current perspectives and how they choose to present themselves, a limitation embedded in the “cross-sectional design”.

However, the participants contributing in the present study are individuals who wished to reflect upon and share narratives contributing to adaptation, which was the scope of the study. The participants are characterized by mean years since injury, ranging from two to thirty-two years’ living with SCI when sharing experiences on their adaptation processes. The narrative descriptions focused on the importance of personal strength, social relations ability and supportive network and give the impression of participants with resources. However, exploring personal narratives depend on the research question raised, and in the present study the semi structured interview explored a resilience oriented perspective, probably influencing the narratives presented. With other research question raised in terms of a perspective other than exploring resilience or a sample with other participants’ characteristics than in current sample, we might have found other results in the process of adjustment over time. The sample size was also limited in the sense that only men responded to the invitation to in-depth interviews, which calls for the results to be interpreted with caution. The lacks of female participants with SCI is a major limitation of this study and provide uncertainty if the findings also apply to woman. There are limited studies in gender differences in the SCI population; still there is some previous research that has pointed out that resilience is not significantly influenced by gender [26] and that women with SCI reported higher subjective well-being related to interpersonal relations than men [45, 46]. On the other hand, women also report lower satisfaction with health and lower subjective well – being than men [46]. Moreover, the interviews were conducted by professionals from the rehabilitation hospital to which the participants had been admitted. Some participants were therefore familiar with the interviewer, and this may have affected their willingness to convey critique. The strength of the study was the exploration of the long-term experiences of living with SCI.

## Conclusions

Qualitative studies examining the personal experiences of living with SCI in a long-term perspective within the framework of resilience are few. In line with previous studies, our findings highlight protective factors and efforts that may promote and sustain wellbeing across time post-injury in persons with SCI. These may be tools for helping them bounce back when exposed to emotionally stressful events. For instance, participants placed particular focus on the attributions of their innate personal abilities, such as maintaining a positive attitude, staying active and making efforts to live independent and normalised lives.

In addition, many described their strong connection to their families and friends and how they in various ways put considerable effort into adjusting to changed expectations in order to maintain these important relations with altered functional capacities. In addition, the current study contributes nuances and depth that expand our understanding of the construct of resilience. The main original finding of this study is how the participating men with SCI try hard to continuously adjust and adapt to on-going and new challenges. The participants highlighted the importance of exerted agency, willpower and strength by the use of rational cognitive strategies to counteract negative feelings and attributions. Of interest, they described inner dialogues with positive self-talk, analysing the cost–benefits before making decisions, and sorting out their priorities to a greater extent than before the injury.

## Additional file

Additional file 1: Interview guide. (ODT 7 kb)

## Abbreviation

SCI: Spinal cord injury
