# Supplementary material for: Exploring narratives of resilience among seven males living with spinal cord injury: a qualitative study
Source: BMC Psychol. 2018 Jan 4;6:1. doi: 10.1186/s40359-017-0211-2 (PMC5755441; doi:10.1186/s40359-017-0211-2)
Supplement: Supplementary file 1 [file 40359_2017_211_MOESM1_ESM.odt]

The guide for in-depth interview

The interviews will be digitally recorded. The guide contains of core questions to the research themes. The main focus of this project is: «what are the experiences the family members have after traumatic injuries in a resilience perspective»

The individual in-depth interviews will focus on the participants` life history and how previous experiences contribute in their meeting with current pressures in their lives. What thoughts and feelings do the interviewees have of that which has happened, and how do they interpret and understand it in the light of their own life history and experiences? Which previous experiences from their life can they utilize to help them, and what must they work through in a new and changed situation? Does what has happened have any reconciliatory aspects, or is the situation primarily characterized by loss and grief where one struggles to find direction? Does the persons experience that there is “a light at the end of the tunnel” because unity, strength, hope and courage help them.  Do they have morals of life, values and characteristics they believe are of importance in tackling considerable pressures?

These interviews will emphasize the participants` narratives about the processes their family has been through after one family member suffered a spinal injury or brain injury.  In addition we will ask how their life history has been of importance for their understanding of the situation and their function as support person for people with serious injuries

Background:

#### -Tell me about yourself and your family; what were you and your family like before the injury occurred?

-What experiences do you have of tackling pressures of life? What

 about the others in your family?

-Which life strains would you say have been of importance to you? What will you say has helped you when faced with demanding situations? (personal characteristics, environment, social support, anything else?)

-Did you ever experience that you fell short or were lacking

necessary tools? Did facing major life challenges teach you anything that effected your character which you may make use of now?

Can you describe the process you and your family has been through since the injury occurred

-What do you think your role has been after one of your nearest was seriously injured?

-Would you say that anything has changed for you, on a personal level, throughout what you       have experienced?

-Has your family experienced changes and grown with the challenges?

Which characteristics do you feel have contributed positively in your family and for you? What have you had to help yourselves with in such a challenging situation?

-How does the family use each other to tackle the challenges you 	experience?

-What do you feel are the greatest challenges you face today?

What do you think has been of importance for your family during this time?

-Has your family had norms and values that have been of assistance to you in such a challenging situation? Has your family or you as person had stability which you could use to give hope and faith, counteracting pessimism and fear?

-Have hope and optimism been important for giving you support? In what way? (During rehabilitation – the hope of regaining function – other forms of hope)

-What have your needs been for gaining information, practical adaptations and support?

-What have you missed after the experience of having a serious injured person within the family? Is there anything you feel has failed? (For example providing assistance from experts)

What is of importance to you today?

What can improve the situation?

How do you view the future in the light of what your family has experienced?

Is there anything else which you feel is important that has not been mentioned?

Thank you for sharing your experiences with us.
